# Psychometric properties of the Persian version of the food choice questionnaire and food choice motives among the study population

**DOI:** 10.1017/S1368980022002233

**Published:** 2023-03

**Authors:** Sara Jalali-Farahani, Parisa Amiri

**Affiliations:** Research Center for Social Determinants of Health, Research Institute for Endocrine Sciences, Shahid Beheshti University of Medical Sciences, Tehran, Iran

**Keywords:** Food choice, Validity, Reliability, Socio-demographic factors, Adults, Iran

## Abstract

**Objective:**

This study aimed to evaluate the psychometric properties of the Persian version of the food choice questionnaire (FCQ) and determine food choice motives among different study subgroups.

**Design:**

This cross-sectional study was conducted using self-administered questionnaires, including socio-demographic information and body weight and height data. In addition, study samples were asked to complete the Persian version of the FCQ.

**Setting:**

Educational and medical centres under the coverage of the Shahid Beheshti University of Medical Sciences in Tehran.

**Participants:**

Study samples were 871 adults (60·5 % female) selected using a convenience sampling method.

**Results:**

Mean ± sd age and BMI were 33·4 ± 10·7 years and 24·3 ± 5·2 kg/m^2^, respectively. More than one-third of the study samples were overweight/obese (35·8 %). A nine-structure model including thirty-two items of the original FCQ showed acceptable fit indices as follows: χ^2^/df = 3·39, goodness-of-fit index = 0·905, incremental fit index = 0·92, comparative fit index = 0·92, root mean square error of approximation (90 % CI) = 0·052 (0·049, 0·055). Regarding food choice motives, the three most important motives for food choice ranked by study samples were sensory appeal, natural content and health, respectively. Study samples ranked ethical concern as the least important food choice motive.

**Conclusion:**

These findings support the reliability and validity of the Iranian version of the FCQ. Additionally, results indicate the most important motives for food choice across various socio-demographic and weight status groups which can provide beneficial information for marketing practices in Iran and promote the food choices of Iranians.

Food choice has been defined as a ‘set of decisions made subconsciously or consciously by a person at the time of purchase, during food consumption, or any time between these two’^(1)^. Food choices influences are not confined to the health and well-being of people; what individuals choose to eat can influence agriculture, the environment, business, culture and society’s economy at different levels^(2)^. The food choice questionnaire (FCQ) is a multidimensional scale developed by Steptoe *et al.* to investigate the motives of food choice among different populations. This scale consists of thirty-six items that assess the main motives for food choice across nine subscales: health, mood, convenience, sensory appeal, natural content, price, weight control, familiarity and ethical concern^(3)^. This scale has been used in different populations worldwide, including in European (Germany, Italy, Greece, Ireland, Poland, Bosnia-Herzegovina, Croatia, Montenegro, Serbia, Slovenia, Portugal, Spain, the Netherlands, the UK and Norway)^(4–6)^, Asia and Oceania (South Korea, Japan, Taiwan, Malaysia and New Zealand)^(7,8)^, North and South America (USA, Canada, Brazil and Uruguay)^(6,9–11)^ and African (South Africa and Cape Verde) countries^(12,13)^. Although the nine-factor structure of the original FCQ was invariant in several previous studies, its nine-factor structure has not been confirmed in all studied populations^(14)^. In this regard, some adaptations were made, and a revised version of the FCQ was introduced. For example, in a number of previous studies, rearrangement of items was applied^(12)^. In studies conducted in Italian, Belgian and Balkan countries’ samples, health and natural content subscales were included in a single factor^(5,6)^. In other studies conducted in Brazil and Greece, the ethical concern subscale has been removed from the revised version^(10,15)^. Nevertheless, applying a modified version of this scale across different countries is still beneficial. It provides the chance for a cross-cultural comparison of food choice motives in various parts of the world.

Iran, as a country experiencing a nutritional transition, have been encountered significant changes in individuals’ dietary patterns^(16)^. The results of a review showed that the per capita calorie intake in Iran was more than the recommended daily allowance; in addition, per capita consumption of oil and sugar was 20 and 38 % more than the recommended amounts in the standard food basket, respectively^(17)^. These findings highlight the necessity of modifying food choices in the Iranian population. Since different factors can influence people’s food choices, identifying the influential factors is essential for planning efficient public policies and designing appropriate interventions targeting healthier eating habits in different age and socio-demographic groups.

In the UK, where the FCQ was first used, health, convenience and sensory appeal were the factors most emphasised by consumers than any other food choice motives^(18)^. A similar study conducted in Russia showed that sensory appeal and availability, followed by price, were the most important factors influencing food choice^(19)^. In another study, food choice motives were assessed in Japan, Taiwan, Malaysia and New Zealand. The results showed that motives for food choice are strongly related to nationality^(7)^. Given the differences in food choice motives in different countries and cultures, identifying racial and cultural differences in food choice motives seems essential. The FCQ has been used across many countries and examines food selection motives from different dimensions. In addition, completing this questionnaire is easy and does not require much time. These characteristics make it an appropriate tool for assessing food choices in large populations for cross-cultural comparisons. Since this questionnaire has not been validated in Iran; therefore, the present study has been conducted to evaluate the validity and reliability of the FCQ in Iranian adults.

## Methods

### Study samples

This cross-sectional study was conducted among 1077 adults ≥ 18 years. There is no internationally accepted method for determining the sample size for confirmatory factor analysis (CFA), and various guidelines exist for calculating sample sizes for CFA. It is mentioned that less than 100 is considered ‘small’ and may only be appropriate for very simple models; 100 to 200 is ‘medium’ and maybe an acceptable minimum sample size if the model is not too complex and greater than 200 is ‘large,’ which is probably adequate for most models^(20)^. Moreover, when the number of indicators per factor in CFA models is more than 2, it is necessary to consider increasing the sample size to more than 400^(21,22)^. Considering another aim of the current study regarding comparing food motives across various study subgroups such as socio-demographic groups, more than 1000 individuals completed the Persian version of FCQ to meet an adequate sample size in each subgroup after excluding individuals with chronic diseases. Study samples were selected using a convenience sampling method from educational and medical centres under the supervision of the Shahid Beheshti University of Medical Sciences. As different diseases may influence the food choice of individuals, before data analysis, those with a history of chronic diseases were excluded; hence, a total of 871 individuals (60·5 % female) remained in data analysis.

### Measurements

After providing a brief explanation regarding the aim of the study and the way of completing the questionnaire, study samples were asked to complete a set of questions regarding socio-demographic variables such as age, sex, marital status, education, job, income and household size. In addition, they were asked to self-report their weight, height and history of diseases. BMI was calculated as weight (kg) divided by the square of height (m^2^). The weight status of study samples was determined using their BMI as follows: (1) underweight: BMI < 18·5 kg/m^2^, (2) normal weight: BMI of 18·5 to < 25 kg/m^2^, (3) overweight: BMI of 25 to < 30 kg/m^2^ and (4) obese: BMI ≥ 30 kg/m^2^).

The study samples were also asked to fill out the Persian version of the FCQ. The original version of the FCQ encompasses 36 items which are developed by Steptoe *et al.* (1995) and measures the main motives for food choice in nine categories, including (1) health (6 items), (2) mood (6 items), (3) convenience (5 items), (4) sensory appeal (4 items), (5) natural content (3 items), (6) price (3 items), (7) weight control (3 items), (8) familiarity (3 items) and (9) ethical concern (3 items). A four-point Likert scale (ranging from 1 = not important at all to 4 = very important) has been used for scoring the questionnaire.

### Translation

For linguistic validation and translation of the FCQ into the Persian language (Iranian), the recommended guidelines were followed. First, permission has been sought to translate and use the questionnaire from the FCQ developer (Prof. Andrew Steptoe). To conduct forward translation, the FCQ was independently translated from English to Persian by two experts in nutrition science and health education. Both translators discussed discrepancies and agreed on a single version to provide a conceptually equivalent translation of the original FCQ. Then, the final version of the FCQ was back-translated to English by a professional translator who was familiar with both the Persian and English languages and had experience living in English-speaking countries. After this stage, the backward translation was sent to the FCQ developer for any required modifications, and after the revision of the FCQ by the developer, necessary corrections were made.

### Statistical analysis

Data analysis was done using the SPSS and AMOS graphic version 21 software. To describe continuous and categorical variables, mean ± sd and frequency (%) were used, respectively.

### Validity

A qualitative method was used to assess face validity to ensure the difficulty level, the degree of appropriateness and the ambiguity of questions. Moreover, for qualitative content validity, the experts were asked to express their views on the observance of language grammar and the scale scoring. Any essential corrections were made according to the expert panel’s comments before completing the questionnaire by the study samples.

A CFA with a maximum likelihood method was performed to determine the structural validity of the Persian version of the FCQ. Model fit indices such as χ^2^/df, root mean square error of approximation and its 90 % CI, comparative fit index, incremental fit index and goodness-of-fit index were used to assess the fitness of the nine-factor CFA model. For χ^2^/df, there is no consensus on an acceptable value for this statistic. Based on recommendations, acceptable values range from 2·0 to 5·0, and values < 3 is considered an indicator of good model fit. For root mean square error of approximation, values < 0·08 and close to 0·06 indicate a good model fit. Comparative fit index, incremental fit index and goodness-of-fit index values indicate acceptable and good model fit at values > 0·90 and > 0·95, respectively^(20,23)^.

### Reliability

Internal consistency was assessed using Cronbach’s alpha coefficients for the whole FCQ scale and its subscales, and *α* values greater than 0·7 were considered acceptable^(24)^. The intra-class correlation coefficients (ICC) were employed to assess test–retest reliability. For this purpose, the FCQ was completed by thirty adults within a 14-d interval. The ICC were calculated for items and subscale scores of the two tests, and ICC values greater than 0·5 were considered acceptable.

## Results

### Descriptive statistics

Table 1 shows the descriptive statistics of the study samples. The mean ± sd age and BMI were 33·4 ± 10·7 years and 24·3 ± 5·2 kg/m^2^, respectively. Approximately half of the study samples were married (49·9 %). Males and females had significantly different distributions of job status (*P* < 0·001). A higher proportion of men were employed compared to women, and a higher proportion of women were a student or household/retired compared to men. In addition, a higher percentage of males were overweight/obese compared to females (49·1 *v*. 27·0%).

**Table 1:** Descriptive statistics of study samples

	Total (*n* 871)		Men (*n* 344)		Women (*n* 527)		*P* value
	*n*	%	*n*	%	*n*	%	
Age (*n* 853)							
Mean	33·5		33·9		33·2		0·322
sd	10·7		11·3		10·4		
Marital status (*n* 866)							
Married	432	49·9	177	52·1	255	48·5	
Single	416	48·0	162	47·6	254	48·3	
Widowed/divorced	18	2·1	1	0·3	17	3·3	
Level of education (*n* 869)							
Less than high school diploma	43	5·0	18	5·2	25	4·8	0·577
High school diploma	173	19·9	74	21·5	99	18·8	
Academic degree	653	75·1	252	73·3	401	76·4	
Job-status (*n* 848)							
Employed	440	51·9	220	66·7	220	42·5	< 0·001
Student	244	28·8	83	25·1	161	31·1	
Household/retired	164	19·3	27	8·2	137	26·4	
Household size (*n* 858)							
≤ 3 members	403	47·0	166	49·3	237	45·5	0·262
4 members and more	454	53·0	170	50·7	284	54·5	
Family income (*n* 825)							
< 30 million Rials	271	32·9	122	36·6	149	30·3	0·161
30–50 Million Rials	246	29·8	93	27·9	153	31·1	
> 5 million Rials	308	37·3	118	35·4	190	38·6	
Residential area (*n* 810)							
Tehran	650	80·3	248	77·5	402	82·0	0·113
Other (suburban areas)	160	19·7	72	22·5	88	18·0	
Body weight status (*n* 837)							
Underweight	32	3·8	10	3·0	22	4·4	< 0·001
Normal weight	505	60·3	162	48·1	343	68·6	
Overweight and obese	300	35·9	165	49·1	135	27·0	

### Validity of the food choice questionnaire

The CFA was conducted to evaluate the structural validity of the Persian version of the FCQ. Standardised factor loadings for all items ranged between 0·50 and 0·83 (greater than 0·40). The fit indices for nine-factor structure of the original model were as follows: χ^2^/df = 4·76, goodness-of-fit index = 0·84, incremental fit index = 0·86, comparative fit index = 0·86, root mean square error of approximation (90 % CI) = 0·066 (0·063, ,0·068) and *P*-value < 0·001. Considering that the fit indices for this stage did not have optimal values; hence, we tried to achieve an optimal fit by conducting modifications with minimal changes in the original questionnaire. For this purpose, we tried to keep the nine-factor structure without changing subscales (removing, adding or merging). Instead, we applied more minor changes, such as changing items to achieve an acceptable fit. Therefore, to increase the structural validity of the questionnaire and improve the fit indices, we tried removing items whose removal from the questionnaire did not damage the concept of food choice; all of them had corresponding questions in their subscale. Additionally, various internal factor error covariance modifications were performed. After removing items number 1, 17, 18 and 34 from the original questionnaire, a total of thirty-two items were kept. In the refined nine-factor thirty-two-item model, nine sets of item error covariance correlations were considered in the model. These error covariance correlations between each pair of items were as follows: items 9 and 10, items 10 and 22, items 29 and 30, items 16 and 26, items 11 and 35, items 15 and 28, items 2 and 23, items 6 and 36 and items 20 and 32. Finally, the nine-structure thirty-two-item model showed acceptable fit indices as follows: χ^2^/df = 3·39, goodness-of-fit index = 0·905, incremental fit index = 0·92, comparative fit index = 0·92 and root mean square error of approximation (90 % CI) = 0·052 (0·049, 0·055).

### Reliability of the food choice questionnaire

Mean and sd, factor loadings, ICC for items and Cronbach’s alpha for subscales of the thirty-two-item FCQ are presented in Table 2. The ICC values for subscales of the FCQ ranged from 0·83 to 0·93 and were as follows: health (ICC = 0·873), mood (ICC = 0·914), convenience (ICC = 0·825), sensory appeal (ICC = 0·850), natural content (ICC = 0·932), price (ICC = 0·863), weight control (ICC = 0·856), familiarity (ICC = 0·911) and ethical concern (ICC = 0·852). Moreover, the alpha value for overall items of the FCQ was 0·92; for subscales, the alpha ranged from 0·68 to 0·88.

**Table 2:** Mean and sd, factor loadings, intra-class correlation coefficients (ICC) for items and Cronbach’s alpha (*α*) for subscales of the food choice questionnaire

Subscales	Items	Mean	sd	Standardised factor loadings	ICC	*α*
Health	Is high in fibre and roughage	2·78	0·94	0·68	0·799	0·88
	Is nutritious	3·06	0·85	0·75	0·818	
	Contains lots of vitamins and minerals	3·08	0·89	0·81	0·765	
	Is high in protein	2·91	0·91	0·62	0·556	
	Keeps me healthy	3·33	0·83	0·78	0·800	
	Is good for my skin/teeth/hair/nails etc.	3·05	1·00	0·76	0·856	
Mood	Cheers me up	3·17	0·87	0·71	0·777	0·83
	Helps me cope with stress	2·58	1·06	0·70	0·769	
	keeps me awake and alert	2·71	1·00	0·63	0·676	
	Helps me relax	2·84	0·98	0·78	0·874	
	Makes me feel good	3·10	0·91	0·68	0·737	
Convenience	Is easily available in shops and supermarkets	2·72	0·95	0·66	0·723	0·76
	Can be cooked very simply	2·86	0·93	0·57	0·850	
	Takes no time to prepare	2·80	0·94	0·50	0·820	
	Can be bought in shops close to where I live or work	2·74	0·96	0·69	0·596	
Sensory appeal	Tastes good	3·66	0·66	0·56	0·833	0·70
	Smells nice	3·27	0·82	0·75	0·822	
	Looks nice	3·08	0·87	0·67	0·780	
Natural content	Contains no additives	2·84	1·02	0·68	0·858	0·80
	Contains natural ingredients	3·30	0·81	0·76	0·875	
	Contains no artificial ingredients	3·05	0·96	0·79	0·739	
Price	Is not expensive	2·74	0·90	0·82	0·609	0·88
	Is good value for money	2·90	0·91	0·87	0·861	
	Is cheap	2·64	0·94	0·83	0·818	
Weight control	Is low in calories	2·53	1·01	0·68	0·828	0·71
	Is low in fat	3·00	0·94	0·81	0·706	
Familiarity	Is familiar to me	2·51	0·99	0·66	0·757	0·68
	Is like the food I ate when I was a child	2·03	0·98	0·57	0·831	
	Is what I usually eat	2·41	0·91	0·73	0·841	
Ethical concern	Is packaged in an environmentally friendly way	2·50	1·09	0·72	0·891	0·69
	Comes from countries I approve of politically	1·64	0·98	0·48	0·707	
	Has the country of origin clearly marked	2·23	1·14	0·65	0·804	

### Correlations between subscales

Table 3 shows the correlations between the FCQ subscales, indicating the extent to which the subscales measure related subconstructs. These correlations are ideal to be moderate (*r* = 0·3–0·7) to indicate these different subscales measure related but different subconstructs. In the current study, all correlations between subscales of the FCQ were statistically significant at a 95 % CI level (*P*-value < 0·001) and ranged between 0·167 and 0·899.

**Table 3:** Correlation matrix of the food choice questionnaire subscales

	Health	Mood	Convenience	Sensory appeal	Natural content	Price	Weight control	Familiarity	Ethical concern
Health									
Mood	0·758*								
Convenience	0·439*	0·458*							
Sensory appeal	0·516*	0·766*	0·418*						
Natural content	0·899*	0·592*	0·374*	0·437*					
Price	0·219*	0·259*	0·667*	0·228*	0·227*				
Weight control	0·684*	0·479*	0·362*	0·309*	0·775*	0·274*			
Familiarity	0·350*	0·441*	0·588*	0·416*	0·307*	0·337*	0·167*		
Ethical concern	0·735*	0·747*	0·384*	0·437*	0·685*	0·257*	0·456*	0·537*	

* Shows a significant correlation between subscales at the 95 % CI level.

### The food choice motives

Mean ± sd subscale scores of health, mood, convenience, sensory appeal, natural content, price, weight control, familiarity and ethical concern were 3·03 ± 0·7, 2·88 ± 0·8, 2·78 ± 0·7, 3·34 ± 0·6, 3·06 ± 0·8, 2·8 ± 0·8, 2·7 ± 0·9, 2·3 ± 0·8 and 2·12 ± 0·9, respectively. The motives for food choice of different subgroups are provided in Table 4. As indicated in Table 4, except for the underweight group, the three most important motives for food choice of various subgroups of study samples were similar. They included sensory appeal, natural content and health. Similarly, the two least important motives for food choice did not differ among studied subgroups, except for underweight individuals. However, there were some differences in the rank of importance of other motives for food choice, including mood, convenience, price and weight control, across different subgroups of study samples.

**Table 4:** The rank order of the most to least important motives of food choice for each socio-demographic and weight status groups

	Sex		Marital status		Education		Job status			
	Male	Female	Married	Single/widowed/divorced	High school and less	Academic degree	Employed	Student	Household/Retired	
1*	Sensory appeal	Sensory appeal	Sensory appeal	Sensory appeal	Sensory appeal	Sensory appeal	Sensory appeal	Sensory appeal	Sensory appeal	
2	Natural content	Natural content	Natural content	Natural content	Health	Natural content	Natural content	Health	Natural content	
3	Health	Health	Health	Health	Natural content	Health	Health	Natural content	Health	
4	Price	Mood	Mood	Mood	Price	Mood	Mood	Mood	Mood	
5	Mood	Weight control	Weight control	Price	Mood	Weight control	Convenience	Price	Weight control	
6	Convenience	Convenience	Convenience	Convenience	Convenience	Convenience	Weight control	Convenience	Price	
7	Weight control	Price	Price	Weight control	Weight control	Price	Price	Weight control	Convenience	
8	Familiarity	Familiarity	Familiarity	Familiarity	Familiarity	Familiarity	Familiarity	Familiarity	Familiarity	
9	Ethical concern	Ethical concern	Ethical concern	Ethical concern	Ethical concern	Ethical concern	Ethical concern	Ethical concern	Ethical concern	
	Household size		Residential area		Income			Weight status		
	≤3 members	≥4 members	Tehran	Other	< 30 million Rials	30–50 Million Rials	> 5 million Rials	UW.	NW.	OW./Ob.
1*	Sensory appeal	Sensory appeal	Sensory appeal	Sensory appeal	Sensory appeal	Sensory appeal	Sensory appeal	Sensory appeal	Sensory appeal	Sensory appeal
2	Natural content	Natural content	Natural content	Health	Natural content	Natural content	Natural content	Mood	Natural content	Natural content
3	Health	Health	Health	Natural content	Health	Health	Health	Convenience	Health	Health
4	Mood	Mood	Mood	Mood	Price	Mood	Mood	Health	Mood	Mood
5	Convenience	Price	Convenience	Price	Mood	Price	Weight control	Price	Convenience	Weight control
6	Weight control	Convenience	Weight control	Convenience	Convenience	Convenience	Convenience	Natural content	Price	Price
7	Price	Weight control	Price	Weight control	Weight control	Weight control	Price	Familiarity	Weight control	Convenience
8	Familiarity	Familiarity	Familiarity	Familiarity	Familiarity	Familiarity	Familiarity	Ethical concern	Familiarity	Familiarity
9	Ethical concern	Ethical concern	Ethical concern	Ethical concern	Ethical concern	Ethical concern	Ethical concern	Weight control	Ethical concern	Ethical concern

UW., underweight, NW., normal weight, OW., overweight and Ob., obese.

* Numbers 1 and 9 indicate the rank of the most and the least important factors, respectively.

Further analyses were conducted to investigate any statistical differences in FCQ subscale scores among body weight status groups. The findings indicated there were significant differences in the health, natural content, weight control, familiarity and ethical concern subscale scores among body weight status groups. Findings of post hoc tests indicated that the underweight group had significantly lower scores in health subscales compared to the normal weight (*P* = 0·043) and overweight/obese (*P* = 0·014) groups. Similarly, they had lower scores in the natural content subscale compared to the normal weight (*P* = 0·004) and overweight/obese (*P* = 0·001) groups. Moreover, underweight individuals had lower scores in the weight control subscale compared to normal weight and overweight/obese groups (*P* < 0·001). In addition, the overweight/obese group had significantly higher scores in the familiarity subscale, compared to the normal weight (*P* = 0·001) and underweight (*P* = 0·004) groups, and in the ethical concern subscale, compared to the normal weight group (*P* = 0·004).

## Discussion

This study aimed to first investigate the validity and reliability of the Iranian version of the FCQ and then determine the main food choice motives among different groups of study samples. The current findings indicate that a thirty-two-item FCQ is a valid and reliable questionnaire that can be used in the Iranian population. Moreover, findings show the three first important food choice motives (sensory appeal, natural content and health) were the same among study samples with different socio-demographic characteristics, including sex, marital status, education, job status, household size, residential area and income. Similarly, familiarity and ethical concern were the least important food choice motives among these groups. However, some differences were observed regarding the importance of food choice motives, including mood, convenience, price and weight control, across different subgroups of study samples.

Considering the results of CFA, the structural validity of the nine-factor structure of thirty-two-item FCQ was confirmed. This Iranian version is strongly similar to the original questionnaire developed by Steptoe^(3)^. Considering χ^2^/df values of three or less as acceptable values recommended by scholars^(25)^, in the current study, the χ^2^/df value of 3·39 was the only index that seems to be out of the acceptable range among the model fit indices. It is noteworthy to note that the Chi-square value depends on sample size; for large sample sizes, values of five or less have been considered indicators of reasonable fit by some scholars^(26,27)^. Therefore, considering the large sample size in the current study, a value of 3·39 seems to be an indicator of reasonable fit. We tried to keep the structure of the original questionnaire; hence, there was no change in labelling and rearrangement of the items. However, we had to eliminate a few items (items number 1, 17, 18 and 34) to improve the fit indices of the CFA. Similarly, in some of the related previous studies, a modified version of the FCQ was applied in which a number of items were removed or added^(28–33)^. In addition, for all subscales of the FCQ, the Cronbach’s alpha coefficients exceeded the minimum reliability standard of 0·70 except for familiarity (0·68) and ethical concern (0·69), which were very close to the minimum reliability standard. These findings show a good level of internal consistency for the Iranian version of the FCQ. Moreover, in the current study, the ICC values for all subscales were higher than 0·80, demonstrating a good to excellent test–retest reliability for all subscales. Considering these findings, future studies could use the Iranian version of the FCQ as an appropriate scale for exploring food choice motives across different groups of Iranians.

In the current study, there were significant moderate correlations between subscales of the FCQ. Moreover, strong correlations were observed between six pairs of subscales, including weight control and natural content, mood and sensory appeal, mood and ethical concern, health and ethical concern, natural content and ethical concern, weight control and health and convenience and price subscales. These findings show these subscales measure closely conceptually related contents. The highest correlation between subscales of the FCQ was observed between health and natural content, which is consistent with the study conducted in Turkey and European countries^(4,30,34)^. In addition, there was also a strong correlation between mood and health subscales which is in line with findings of European countries^(4)^.

In terms of food choice motives, in the current study, the sensory appeal was the most important factor that influenced the food choice of all study subgroups. Previous studies indicated similarities and differences in motives of food choices across various countries and cultures. The current findings are in line with the results of studies conducted in Turkey, New Zealand, Romania, Hungary, Philippines, Belgium and Western Balkan countries^(5,7,34,35)^. However, in other countries, the most important motives for food choice were different^(7,12)^. For example, in Cape Verde, a combination of items from health and mood subgroups entitled well-being was found to be the most important motives for food choice^(12)^. Similarly, in Taiwan and Malaysia, health was the most important factor that influenced participants’ food choices^(7)^. While, in Japan, the price has been ranked as the first influential factor in food choice by the Japanese^(7)^. Although sensory appeal, natural content and health were the most important motives for food choice in different groups of study samples in this study, sensory appeal accompanied by mood and convenience were ranked as the most important factors by the underweight group. This variance makes sense as the underweight adults have little concern about their health due to having fewer risk factors for developing chronic diseases such as diabetes, hypertension, metabolic disorders and cardiovascular diseases, compared to their overweight and obese counterparts. Therefore, in main motives for food choice, natural content and health-related factors were replaced by other factors such as mood and convenience.

It was surprising that in the current study, the price factor was ranked relatively low compared to other factors. One explanation could be that data collection was conducted during 2019, prior to the manifestation of economic influences of sanctions in Iran. Furthermore, regarding the least importance of familiarity and ethical concern among food choice motives, our findings are in agreement with those of European studies^(4,35)^. This finding implies that nowadays, familiarity and ethical concerns are less attractive factors for consumers. It seems that people are more inclined to try new foods than used to familiar ones. Moreover, considering global environmental concerns regarding food packaging and the importance of using recyclable and environment-friendly packaging materials, it is recommended consumers must be educated to increase their motivation for making better choices of food packaging when they buy and consume foods.

The current findings provide important information for promoting public health policies to improve the food choices of different groups of Iranians. Moreover, current findings would be beneficial for innovations in future food production and food marketing practices in Iran. The limitations of the current study should also be noted. First, study samples of this study were selected using a convenience sampling method; hence, the limitations of such a recruitment procedure must be considered. Therefore, the study population may not be a good representative of the general population; hence, the current findings should not be generalised to all Iranians. Moreover, the cross-sectional design of the current study precludes causal inferences in the association between socio-demographic factors and food choice motives. Due to modifications considered in the Persian version of FCQ, further investigations are suggested to confirm the structural validity of the thirty-two-item FCQ in other Iranian populations.

In conclusion, current findings support the initial reliability and validity of the Iranian version of the FCQ. Moreover, these results indicate sensory appeal, natural content and health as the most important and familiarity and ethical concern as the least important motives for food choices of Iranians across various socio-demographic groups. This study provides beneficial information for marketing practices in Iran and promoting the food choices of Iranians.
